# The perinatal period should be considered in neonatal acute respiratory distress syndrome: comparison of the Montreux definition vs. the second pediatric acute lung injury consensus conference definition

**DOI:** 10.3389/fped.2023.1216073

**Published:** 2023-09-28

**Authors:** Liting Liu, Yihan Zhang, Yiran Wang, Yu He, Xionghui Ding, Long Chen, Yuan Shi

**Affiliations:** ^1^ Department of Neonatology, National Clinical Research Center for Child Health and Disorders, Ministry of Education Key Laboratory of Child Development and Disorders, Chongqing Key Laboratory of Child Infection and Immunity, Children's Hospital of Chongqing Medical University, Chongqing, China; ^2^ Department of Burn and Plastic Surgery, National Clinical Research Center for Child Health and Disorders, Ministry of Education Key Laboratory of Child Development and Disorders, Children's Hospital of Chongqing Medical University, Chongqing, China

**Keywords:** neonatal ARDS, montreux definition, PALICC-2 definition, perinatal period, newborns

## Abstract

**Background:**

The recently developed Montreux definition for neonatal acute respiratory distress syndrome (ARDS) partially differs from the Second Pediatric Acute Lung Injury Consensus Conference (PALICC-2) definition. Here, we compare the Montreux and PALICC-2 definitions regarding morbidity, mortality, and prognosis of neonatal cases of ARDS in order to evaluate which definition is more appropriate for newborns.

**Methods:**

Neonates admitted to our neonatal intensive care unit between 1 January 2018 and 30 September 2019 who met the Montreux or PALICC-2 definition of neonatal ARDS were retrospectively analyzed (*n* = 472). One comparison was made between application of the Montreux and PALICC-2 definitions to neonates outside the perinatal period (> 7 d after birth). A second comparison was made between a diagnosis of neonatal ARDS within (≤ 7 d of birth) and outside (> 7 d after birth) the perinatal period using the Montreux definition.

**Results:**

No significant differences in morbidity, mortality, severity, therapies, or prognosis were observed between neonates in the extra perinatal group according to the Montreux and PALICC-2 definitions. However, epidemiology, clinical course, and prognosis of neonatal ARDS within the perinatal period did differ from those outside the perinatal period according to the Montreux definition.

**Conclusion:**

Neonates with ARDS within the perinatal period have unique triggers, epidemiology, clinical course, and prognosis, yet a similar pathobiology pattern, to neonates at other ages. Therefore, it may be essential to consider the perinatal period when defining neonatal ARDS.

## 1. Introduction

Acute respiratory distress syndrome (ARDS) was first described by Ashbaugh et al. in 1967 as a syndrome of shortness of tachypnea, hypoxia, and decreased pulmonary compliance (1). Since then, the definition and diagnostic of ARDS criteria have been consistently updated, yet remain controversial. Pediatricians have widely applied the American European Consensus Conference (AECC) definition, or the Berlin definition, to describe and study the epidemiology of ARDS in children. However, both of these definitions of ARDS focus on adult lung injury and have limitations when applied to children, including newborns (2–4).

While adult and pediatric ARDS are similar in pathophysiology, there are significant differences in their risk factors, epidemiology, morbidity, therapies, and prognosis. Accordingly, the Pediatric Acute Lung Injury Consensus Conference (PALICC) published the first definition of ARDS in children in 2015 (5). This definition was also applied to neonates (6). However, with the emergence of a growing body of knowledge regarding pediatric ARDS, including its pathobiology, mechanisms of lung protection, and a new mode of ventilation [high-flow nasal cannula (HFNC)] (7, 8), the Second Pediatric Acute Lung Injury Consensus Conference (PALICC-2) updated the definition in 2023 (9). The updates introduced include: (1) patients with pediatric ARDS should be stratified into severity categories after a period of at least 4 h to improve risk stratification and (2) patients receiving non-invasive mechanical ventilation can also be stratified according to pediatric ARDS severity. In addition, the PALICC-2 definition creates a “possible pediatric ARDS” category to include patients who likely have pediatric ARDS. However, while the PALICC-2 definition represents a great advancement in defining pediatric ARDS, it does not include specific perinatal pulmonary insults. Thus, we propose that a new ARDS definition should be established to specifically evaluate neonates.

In 2017, with advocation from the European Society for Pediatric and Neonatal Intensive Care (ESPNIC) and the European Society for Pediatric Research (ESPR), an international multicenter, multidisciplinary assistance group developed the corresponding diagnostic definition for neonatal ARDS (Montreux definition) (10). This was the first internationally accepted definition for neonatal ARDS. Major differences between the Montreux and PALICC-2 definitions are: (1) the Montreux definition includes manifestations of ARDS in the perinatal period; (2) the Montreux definition requires the presence of bilateral pulmonary infiltrates, while the PALICC-2 definition does not; and (3) the Montreux definition uses an oxygenation index (OI), not an oxygenation saturation index (OSI), to diagnose neonatal ARDS and classify its severity, regardless of whether invasive or non-invasive ventilation is used.

To date, no comparison of the definitions of neonatal ARDS according to the Montreux and PALICC-2 definitions has been reported. Therefore, this retrospective study was conducted to compare the Montreux and PALICC-2 definitions in regard to morbidity, mortality, and prognosis of neonatal ARDS cases in order to evaluate which definition is more appropriate for neonates.

## 2. Materials and methods

### 2.1. Study design

This retrospective study was conducted at Children's Hospital of Chongqing Medical University, China. Neonates admitted to the neonatal intensive care unit (NICU) between 1 January 2018 and 30 September 2019 were reviewed to identify infants who met the Montreux or PALICC-2 definitions of neonatal ARDS. The Ethics Committee of Chongqing Medical University approved this study.

### 2.2. Inclusion criteria

All data were extracted from medical records of the admitted patients. Initially, all of the neonates diagnosed with respiratory failure in the NICU between 1 January 2018 and 30 September 2019 were screened through the platform of Chongqing Medical University. Next, patients aged from birth up to 44 weeks post-menstrual age (for neonates born before 40 gestational weeks) or up to 4 weeks postnatal age (for neonates born after 40 gestational weeks) were selected. Three investigators subsequently assessed the presence of unilateral or bilateral consolidation (the presence of diffuse, irregular opacities, infiltrates, or complete opacification of the lungs) on chest radiographs during hospitalization in the NICU, if there were disagreements, the Delphi method was invoked to include evaluations by additional doctors and radiologists to reach consensus. For each assessment, the same protocol and definitions were adhered to. If deemed compatible, a patient was further screened for hypoxemia (partial pressure of oxygen <0.6) within 24 h of their chest radiograph. Patients meeting either the Montreux or PALICC-2 definition were selected for enrollment in this study.

### 2.3. Exclusion criteria

Neonates with a NICU stay <6 h, incomplete medical records, respiratory failure caused by transient tachypnea of the newborn (TTN), neonatal respiratory distress syndrome (NRDS), or congenital pulmonary malformation were excluded.

### 2.4. Definitions of ARDS

Criteria for the Montreux definition includes: (1) acute onset (within 7 days) from a known clinical insult; (2) pulmonary imaging shows bilateral infiltration; (3) absence of congenital heart disease explaining edema and echocardiography that verifies the origin of edema; and (4) according to the Montreux definition, neonatal ARDS severity is defined as severe (OI ≥16), moderate (8 ≤OI <16), or mild (4 ≤OI <8). Transcutaneous values were allowed in the calculation of oxygenation index when arterial values were unavailable (10).

Criteria for the PALICC-2 definition includes: (1) patients with perinatal-related lung disease are excluded; (2) acute onset (within 7 days) from a known clinical insult; (3) respiratory failure that is not fully explained by heart failure or fluid overload; and (4) pulmonary imaging shows new infiltrates consistent with acute pulmonary parenchymal disease. In addition, infants with neonatal ARDS should be stratified into severity categories at least 4 h after the initial diagnosis. For invasive mechanical ventilation (IMV), neonatal ARDS severity is defined as mild/moderate (4 ≤OI <16 or 5 ≤OSI <12) or severe (OI ≥16 or OSI ≥12). For non-invasive mechanical ventilation (NIV), neonatal ARDS severity is defined as mild/moderate (100 <PaO_2_/FiO_2_ (P/F) ≤300 or 150 <SpO_2_/FiO_2_ (S/F) ≤250), or severe (P/F ≤100 or S/F ≤150) (9).

### 2.5. Definitions of complications

Diagnostic criteria for the following diseases were developed based on the corresponding references: bronchopulmonary dysplasia (BPD) (11), shock (12), sepsis (13), intraventricular hemorrhage (IVH) (14), persistent pulmonary hypertension of the newborn (PPHN) (15), retinopathy of prematurity (ROP) (16), neonatal necrotizing enterocolitis (NEC) (17), and meconium aspiration syndrome (MAS) (18).

### 2.6. Data collection

The following clinical data were obtained from the patients with neonatal ARDS who met the Montreux or PALICC-2 definitions: (1) Demographics: gender, age of onset, birth weight, weight at onset, singletons, intrauterine distress, gestational diabetes, gestational hypertension, gestational cholestasis, amniotic cavity infection; (2) Triggers: sepsis, pneumonia, pulmonary hemorrhage, MAS, perinatal asphyxia, and NEC; (3) Other clinical information: 5 min Apgar score, score for neonatal acute physiology with perinatal extension-II (SNAPPE II); (4) Laboratory investigation: chest radiography, blood gas analysis, ventilator mode and its parameters; (5) Therapies: duration of antibiotic use, inhaled nitric oxide usage, duration of mechanical ventilation, length of stay in the NICU and in hospital, home oxygen therapy after discharge, and total cost of hospitalization; (6) Outcomes: death, complications (including BPD, shock, air leaks, secondary sepsis, IVH, PPHN, ROP, and secondary NEC).

### 2.7. Outcomes

One of the major differences between the Montreux and PALICC-2 definitions of neonatal ARDS is that the PALICC-2 definition does not include perinatal neonates. Therefore, the outcomes were analyzed in relation to the following two aspects. (1) For neonates outside of the perinatal period (> 7 d after birth), morbidity, mortality, complications (i.e., BPD, shock, air leaks, secondary sepsis, IVH, PPHN, ROP, and secondary NEC), and therapies for neonatal ARDS were compared by applying the Montreux and PALICC-2 definitions, respectively; (2) Using the Montreux definition, morbidity, mortality, complications, and therapies for patients with neonatal ARDS inside (≤ 7 d of birth) and outside (> 7 d of birth) the perinatal period were compared.

### 2.8. Statistical analysis

Data analysis was performed using SPSS 26.0 statistical software. Normality of data distribution was tested by applying the Kolmogorov-Smirnov test. For data exhibiting normal vs. non-normal distribution, mean [ ± standard deviation (SD)] values or median [interquartile range (IQR)] values are presented, respectively. Qualitative and categorical data are expressed as numbers and percentages. The Chi-square test, or Fisher's exact test, were applied to categorical data while the Mann-Whitney *U*-test, or *t*-test, were applied to continuous data, respectively. All statistical analyses included two-tailed tests, and *p *< 0.05 was considered to indicate a significant difference.

## 3. Results

### 3.1. Baseline patient characteristics

**Figure 1:**
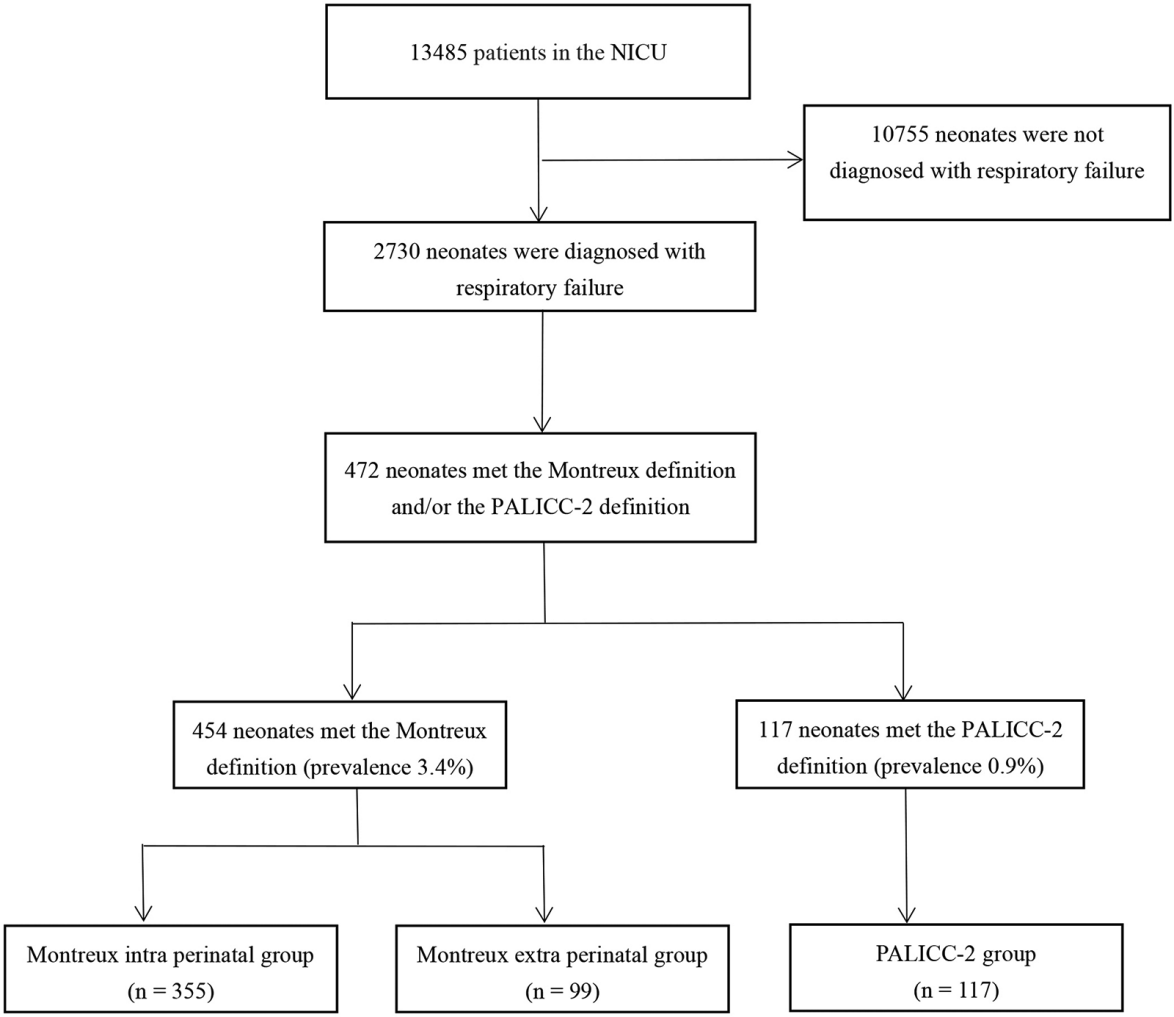
Flow diagram of the study population selected.

**Figure 2:**
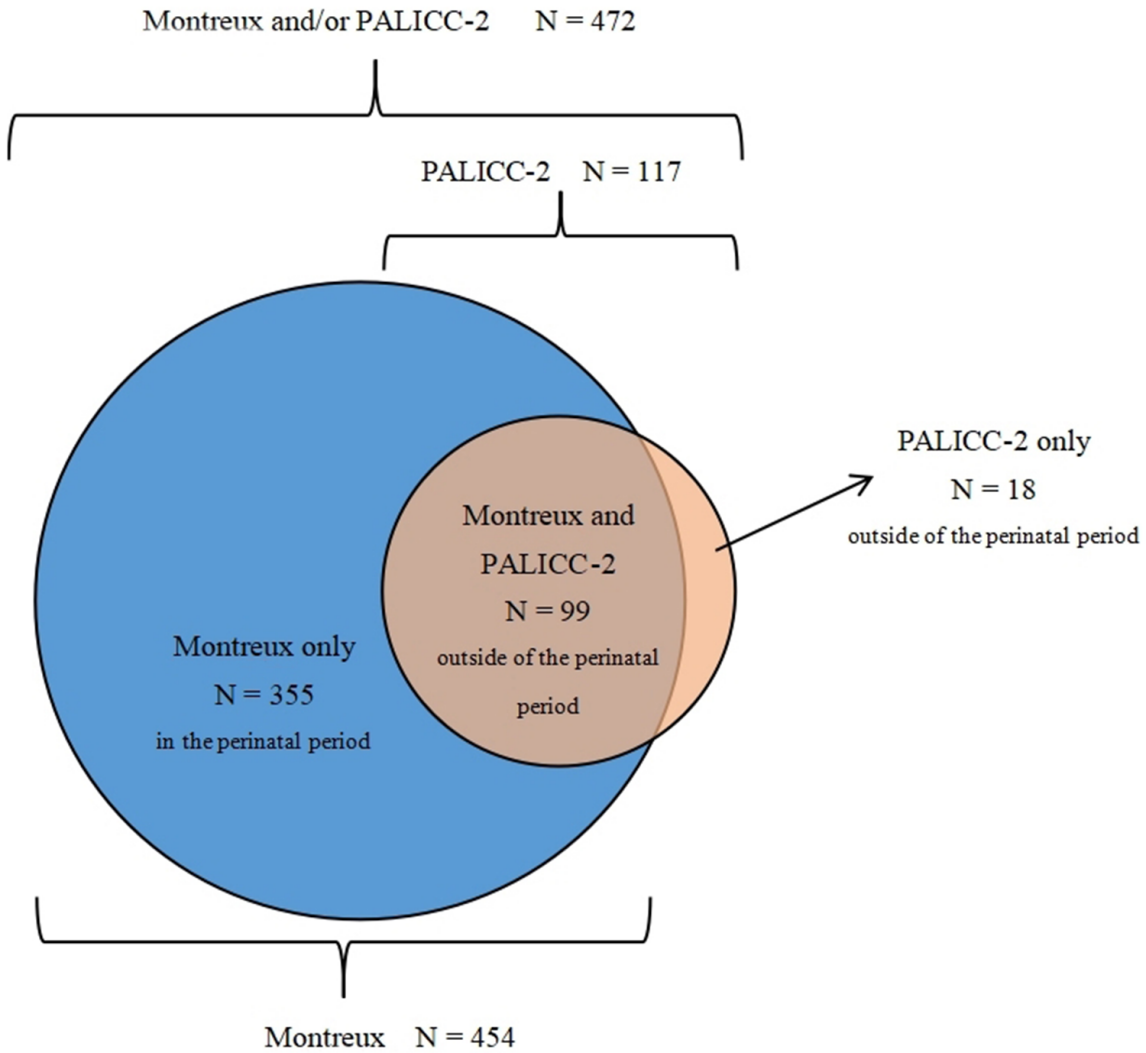
The distribution of patients who met the definition of neonatal ARDS according to the montreux and PALICC-2 definitions. The blue areas represent the 355 neonates who only met the Montreux definition, the dark orange area represents the 99 neonates who met the Montreux and PALICC-2 definitions, and the orange area represents the 18 neonates who only met the PALICC-2 definition.

During the study period, there were 13485 patients admitted to the NICU. Among them, 2,730 neonates were diagnosed with respiratory failure. Of these, 472 neonates met the Montreux definition and/or the PALICC-2 definition and were diagnosed. Severity of neonatal ARDS was graded according to arterial blood gas values. A total of 454 neonates (3.4% of NICU admissions) met the Montreux definition, of whom 99 were outside the perinatal period (> 7 d of birth) at the time of onset. Meanwhile, 355 were within the perinatal period (≤ 7 d of birth). There were 117 neonates (0.9% of NICU admissions) who met the PALICC-2 definition (Figure 1). Of these, 99 neonates were outside the perinatal period and met both definitions, while the remaining 18 neonates only met the PALICC-2 definition (Figure 2). A significant difference in morbidity was observed among the neonates admitted to the NICU for neonatal ARDS according to the two definitions (*p *< 0.001). This difference is primarily attributed to the exclusion of neonates in the perinatal period according to the PALICC-2 definition. Baseline characteristics of the infants examined are summarized in Table 1.

**Table 1:** Characteristics of the patient groups meeting the neonatal ARDS definitions applied.

Variables	Montreux extra perinatal group	PALICC-2 group	Montreux intra perinatal group	*P_1_*value	*P_2_* value
*N*	99	117	355		
Males, *n* (%)	64 (64.6)	71 (60.7)	236 (66.5)	0.549	0.733
Age of onset (days), median (IQR)	22.0 (14.7–33.0)	21.0 (13.0–32.5)	0.13 (0.04–0.96)	0.811	< 0.001
Caesarean delivery, *n* (%)	55 (55.6)	71 (60.7)	281 (79.2)	0.446	< 0.001
5’ Apgar score, median (IQR)	9 (7–10)	7 (7–10)	9 (8–10)	0.469	0.001
Birth weight (g), mean (SD)	1798.7 (893.7)	1856.3 (925.2)	2313.2 (843.9)	0.646	0.432
Weight at onset (g), mean (SD)	2290.7 (951.5)	2342.9 (981.7)	2334.4 (1013.3)	0.705	0.226
Singletons, *n* (%)	74 (74.7)	87 (74.4)	296 (83.4)	0.948	0.050
Gestational age (weeks), median (IQR)	31.4 (28.1–36.7)	31.7 (28.7–36.9)	34.7 (31.9–37.3)	0.657	< 0.001
SNAPPE II score, median (IQR)	16 (5–26)	16 (5–26)	12 (5–16)	0.799	0.003
Mother's age at delivery ≥35 y, *n* (%)	13 (13.1)	16 (13.8)	62 (17.5)	0.887	0.305
Gestational diabetes, *n* (%)	13 (13.1)	17 (14.5)	94 (26.5)	0.767	0.006
Gestational hypertension, *n* (%)	10 (10.1)	10 (8.5)	52 (14.6)	0.695	0.244
Gestational cholestasis, *n* (%)	3 (3.0)	4 (3.4)	18 (5.1)	0.592*	0.291*
Amniotic cavity infection, *n* (%)	1 (1.0)	2 (1.7)	19 (5.4)	0.563*	0.045*
Mode of ventilation at diagnosis					
Invasive, *n* (%)	75 (75.8)	90 (76.9)	273 (76.9)	0.841	0.812
Non-invasive, *n* (%)	24 (24.2)	27 (23.1)	82 (23.1)		
Triggers					
MAS, *n* (%)	0 (0.0)	1 (0.9)	7 (2.0)	0.542*	0.176*
Pneumonia, *n* (%)	47 (47.5)	51 (43.6)	117 (33.0)	0.568	0.008
Pulmonary hemorrhage, *n* (%)	8 (8.1)	11 (9.4)	53 (14.9)	0.733	0.077
Sepsis, *n* (%)	38 (38.4)	45 (38.5)	167 (47.0)	0.991	0.126
Perinatal asphyxia, *n* (%)	3 (3.0)	6 (5.1)	61 (17.2)	0.339*	< 0.001*
NEC, *n* (%)	8 (8.1)	12 (10.3)	1 (0.3)	0.583	< 0.001*

*P1* value: *P* values between the Montreux extra perinatal group and the PALICC-2 group. *P2* value: *P* values between the Montreux extra perinatal group and the Montreux intra perinatal group. SNAPPE-II, score for neonatal acute physiology with perinatal extension-II; PROM, premature rupture of membranes; SD, standard deviation; IQR, interquartile range; MAS, meconium aspiration syndrome; NEC, neonatal necrotizing enterocolitis.

* Fisher's exact test.

### 3.2. Comparison between the Montreux and PALICC-2 definitions for neonates outside the perinatal period

There were no significant differences between the Montreux definition for neonates outside the perinatal period (Montreux extra perinatal group) and the PALICC-2 definition (PALICC-2 group) with regard to baseline characteristics and triggers. The most common trigger was sepsis, followed by pneumonia. Other triggers included: NEC, pulmonary hemorrhage, perinatal asphyxia, and MAS (Table 1).

**Figure 3:**
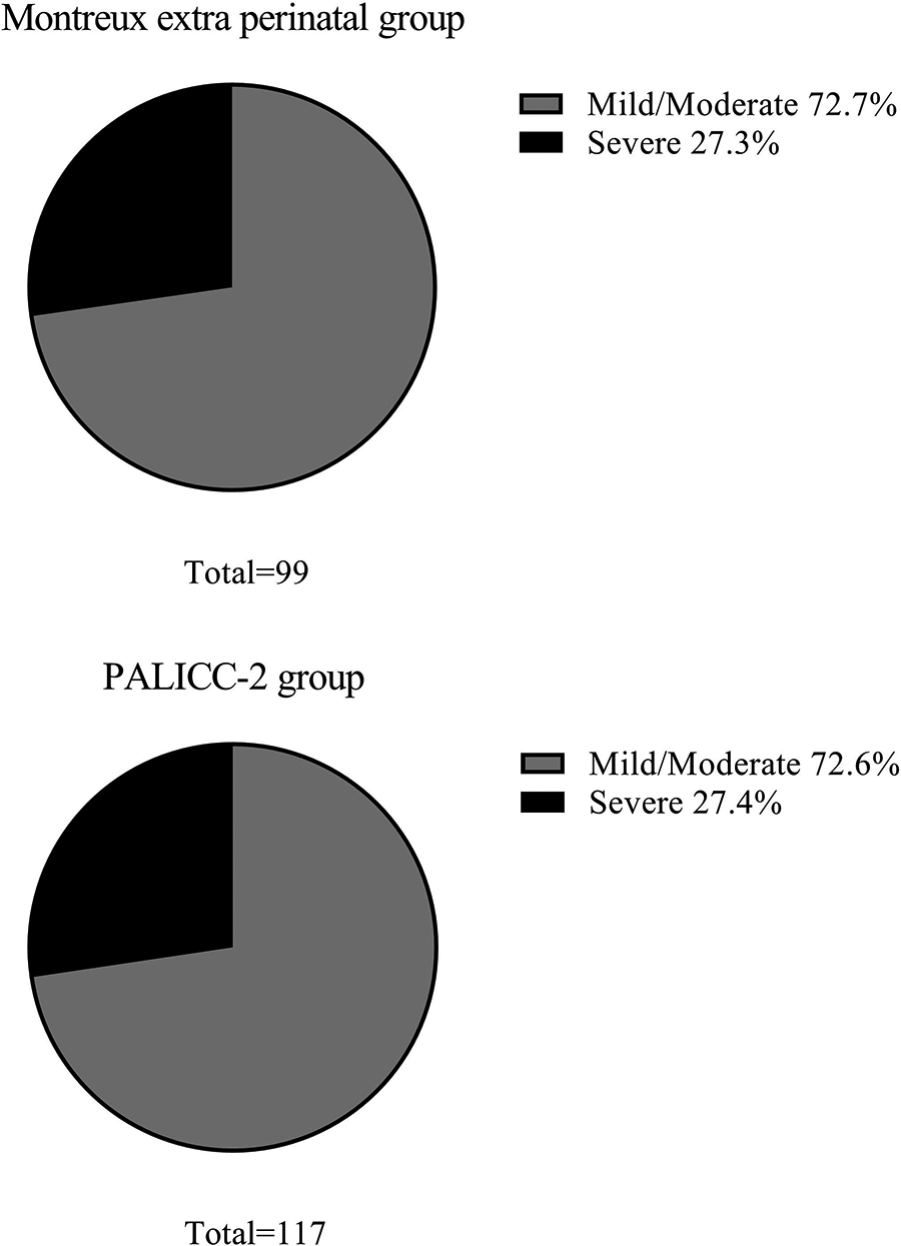
Severity of neonatal ARDS in the montreux extra perinatal group and the PALICC-2 group.

In the Montreux extra perinatal group, 72 infants (72.7%) exhibited mild/moderate ARDS, while 27 (27.3%) experienced severe ARDS. Similarly, the PALICC-2 group included 85 infants (72.6%) with mild/moderate ARDS and 32 infants (27.4%) with severe ARDS. There were no significant differences between the two groups regarding the number of ARDS cases exhibiting various degrees of severity (Figure 3).

There were also no significant differences observed between the two groups in terms of morbidity in the NICU, mortality, complications, and therapies. Furthermore, it was observed that the number of infants in both groups which received home oxygen therapy after discharge did not significantly differ (34.3% *vs*. 31.6%, respectively; *p* = 0.865; Table 2). However, application of the PALICC-2 definition resulted in the identification of an additional 18 patients (22.2% mortality) outside the perinatal period that were not captured by the Montreux definition.

**Table 2:** Comparison between the montreux and PALICC-2 definitions for neonates outside the perinatal period.

Variables	Montreux extra perinatal group (*n* = 99)	PALICC-2 group (*n* = 117)	*P* value
Severity of neonatal ARDS, *n*			
Mild/Moderate, *n* (%)	72 (72.7)	85 (72.6)	0.990
Severe, *n* (%)	27 (27.3)	32 (27.4)	0.990
Death, *n* (%)	27 (27.3)	31 (26.5)	0.898
BPD, *n* (%)	49 (49.5)	52 (44.4)	0.459
Shock, *n* (%)	13 (13.1)	15 (12.8)	0.946
Air leaks, *n* (%)	3 (3.0)	3 (2.6)	0.577*
Secondary sepsis, *n* (%)	20 (20.2)	22 (18.8)	0.796
IVH, *n* (%)	22 (22.2)	25 (21.4)	0.879
PPHN, *n* (%)	7 (7.1)	7 (6.0)	0.746
ROP, *n* (%)	15 (15.2)	16 (13.7)	0.758
Secondary NEC, *n* (%)	5 (5.1)	5 (4.3)	1.000*
Duration of antibiotic use (days), median (IQR)	16.0 (8.0–31)	15.5 (8.08–29.5)	0.831
Inhaled nitric oxide, *n* (%)	10 (10.1)	11 (9.4)	0.140
Duration of invasive ventilator use (hours), median (IQR)	139.0 (47.0–257.0)	141.0 (54.0–260.0)	0.981
Duration of non-invasive ventilator use (hours), median (IQR)	162.0 (45.0–415.0)	133.0 (34.5–383.5)	0.488
Length of stay in the NICU (days), median (IQR)	29.0 (13.0–55.0)	26.0 (12–54.5)	0.687
Length of stay in the hospital (days), median (IQR)	29.0 (14.0–55.0)	26.0 (13.5–54.5.0)	0.738
Home oxygen therapy after discharge, *n* (%)	34 (34.3)	37 (31.6)	0.865
Total cost of hospitalization (US$), median (IQR)	8900.9 (4278.7–15228.2)	8532.3 (4257.5–15003.2)	0.721

ARDS, acute respiratory distress syndrome; BPD, bronchopulmonary dysplasia; IVH, intraventricular hemorrhage; PPHN, persistent pulmonary hypertension of the newborn; ROP, retinopathy of prematurity; NEC, neonatal necrotizing enterocolitis; IQR, interquartile range; NICU, neonatal intensive care unit.

* Fisher's exact test.

### 3.3. Comparison between the infants with neonatal ARDS within and outside the perinatal period according to the Montreux definition

There were no statistically significant differences among a subset of the basic characteristics examined (i.e., gender, birth weight, singletons, gestational hypertension, gestational cholestasis, et al) between the neonates outside the perinatal period (Montreux extra perinatal group) and the neonates within the perinatal period (Montreux intra perinatal group). However, compared to the Montreux extra perinatal group, the Montreux intra perinatal group had a higher proportion of cesarean delivery events (79.2% *vs.* 55.6%, respectively; *p *< 0.001) and gestational diabetes cases (26.5% *vs.* 13.1%, respectively; *p *= 0.006). Likewise, the 5 min Apgar scores and SNAPPEII scores significantly differed between the two groups (*p *< 0.05). In addition, sepsis was the most common trigger in the Montreux intra perinatal group, yet there were no differences compared with the Montreux extra perinatal group. However, compared with the Montreux extra perinatal group, a higher proportion of perinatal asphyxia, and a lower proportion of NEC, were observed among the triggers identified in the Montreux intra perinatal group.

**Figure 4:**
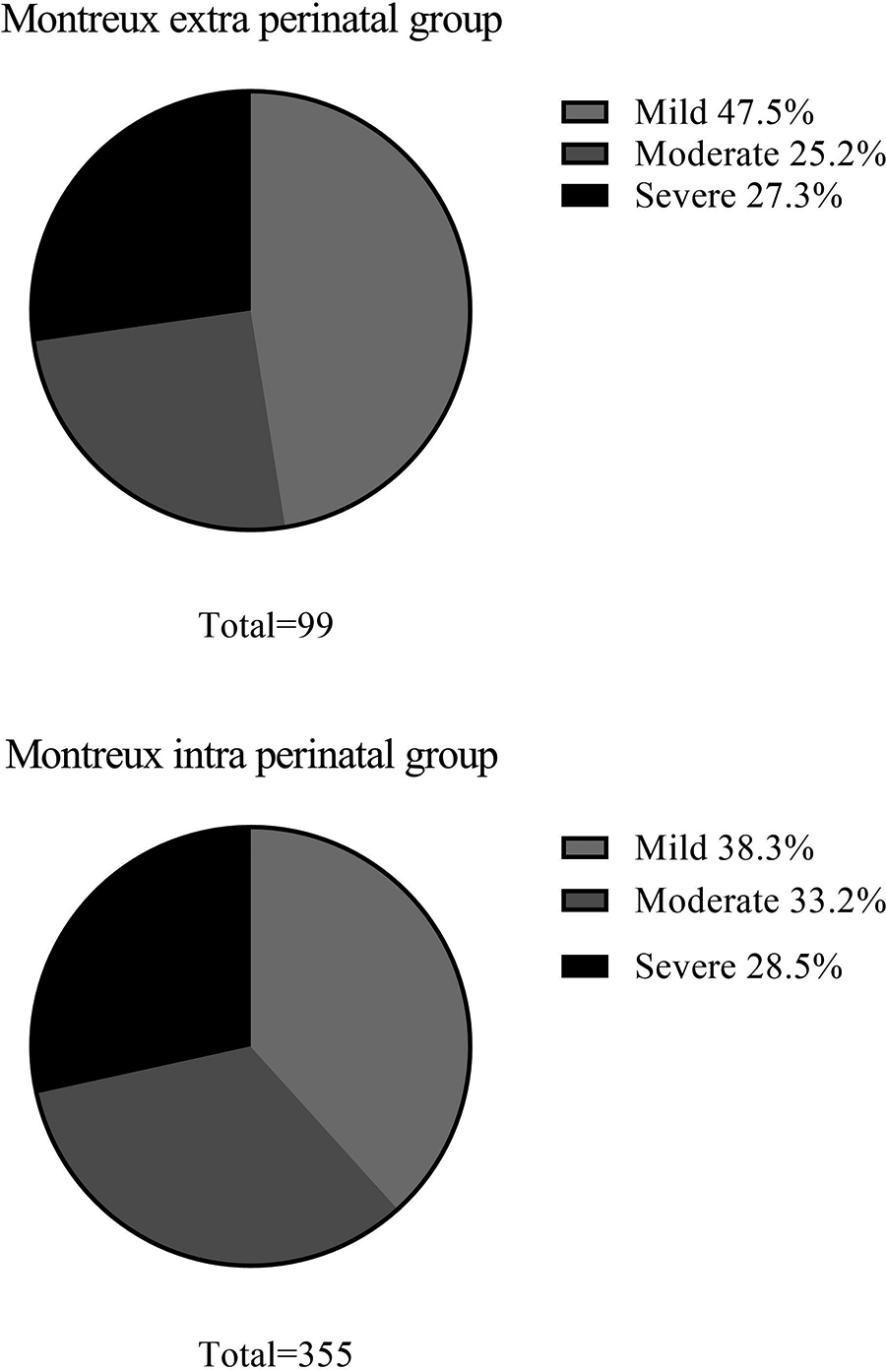
Severity of neonatal ARDS in the montreux extra perinatal group and the montreux intra perinatal group.

The constituent ratio of mild ARDS, moderate ARDS, and severe ARDS cases were found to be similar between the Montreux extra perinatal group and the Montreux intra perinatal group (Figure 4). In addition, BPD was the most common complication in both groups. In contrast, neonate mortality in the Montreux intra perinatal group was significantly lower than in the Montreux extra perinatal group (15.8% *vs.* 27.3%, respectively; *p* = 0.009). Meanwhile, a statistically significant difference in the proportion of partial complications between the two groups were confirmed. For example, compared with the Montreux extra perinatal group, incidence rates of BPD, secondary sepsis, and ROP in the Montreux intra perinatal group were lower (*p *< 0.05), while the rates of air leaks and PPHN were higher (*p *< 0.05) (Table 3).

**Table 3:** Comparison between the infants with neonatal ARDS within and outside the perinatal period according to the montreux definition.

Variables	Montreux extra perinatal group (*n* = 99)	Montreux intra perinatal group (*n* = 355)	*P* value
Severity of neonatal ARDS, *n*			
Mild, *n* (%)	47 (47.5)	136 (38.3)	0.100
Moderate, *n* (%)	25 (25.2)	118 (33.2)	0.130
Severe, *n* (%)	27 (27.3)	101 (28.5)	0.394
Death, *n* (%)	27 (27.3)	56 (15.8)	0.009
BPD, *n* (%)	49 (49.5)	87 (24.5)	< 0.001
Shock, *n* (%)	13 (13.1)	62 (17.5)	0.305
Air leaks, *n* (%)	3 (3.0)	58 (16.3)	< 0.001*
Secondary sepsis, *n* (%)	20 (20.2)	36 (10.1)	0.007
IVH, *n* (%)	22 (22.2)	63 (17.7)	0.313
PPHN, *n* (%)	7 (7.1)	75 (21.1)	0.001
ROP, *n* (%)	15 (15.2)	25 (7.0)	0.012
Secondary NEC, *n* (%)	5 (5.1)	12 (3.4)	0.304*
Duration of antibiotic use (days), median (IQR)	16.0 (8.0–31)	13.0 (8.0–20.0)	0.049
Inhaled nitric oxide, *n* (%)	10 (10.1)	57 (16.1)	0.140
Duration of invasive ventilator use (hours), median (IQR)	139.0 (47.0–257.0)	110.0 (58.0–177.0)	0.039
Duration of non-invasive ventilator use (hours), median (IQR)	162.0 (45.0–415.0)	93.0 (31.0–212.0)	0.019
Length of stay in the NICU (days), median (IQR)	29.0 (13.0–55.0)	21.0 (11.0–38.0)	0.004
Length of stay in the hospital (days), median (IQR)	29.0 (14.0–55.0)	22.0 (13.0–40.0)	0.016
Home oxygen therapy after discharge, *n* (%)	34 (34.3)	56 (15.8)	0.009
Total cost of hospitalization (US$), median (IQR)	8900.9 (4278.7–15228.2)	7793.9 (4858.5–12689.2)	0.204

ARDS, acute respiratory distress syndrome; BPD, bronchopulmonary dysplasia; IVH, intraventricular hemorrhage; PPHN, persistent pulmonary hypertension of the newborn; ROP, retinopathy of prematurity; NEC, neonatal necrotizing enterocolitis; IQR, interquartile range.

* Fisher's exact test.

Regarding therapies for the two groups, duration of antibiotic use, duration of ventilator use, length of stay in the NICU and hospital, and home oxygen therapy after discharge were lower in the Montreux intra perinatal group than in the Montreux extra perinatal group (*p *< 0.05). However, no significant differences were observed between the two groups regarding the rate of inhaled nitric oxide treatment and hospitalization expenses (Table 3).

## 4. Discussion

ARDS refers to severe acute respiratory failure that can be caused by severe infections, trauma, or other reasons. It is characterized by an acute-onset hypoxic condition with radiographic lung infiltration that is associated with a high mortality rate. Previous studies have reported that the morbidity of neonatal ARDS ranges from 1%–5% of NICU admissions, and the mortality rate varies from 17%–24% (19, 20). Survivors may experience substantial physical and neurocognitive disorders, which significantly impair their quality of life (21–23). Similar to pediatric and adult ARDS, neonatal ARDS is associated with high mortality and poor prognosis. If neonatal ARDS is diagnosed, many therapies could be applied earlier, including: PEEP, tidal volume strategies, and modified oxygenation. Early application of adjuvant therapies such as pulmonary surfactant, antibiotics, and inhaled nitric oxide usage may also be considered (24). A standardized definition of neonatal ARDS would help neonatologists conduct basic and clinical studies, and would also further improve our understanding of neonatal ARDS epidemiology. Thus, it is recommended that neonatologists should have greater awareness regarding neonatal ARDS.

As demonstrated in the present study, inclusion of perinatal patients in the Montreux definition substantially increases the number of patients diagnosed with neonatal ARDS compared to the PALICC-2 definition. As a result, use of the Montreux or PALICC-2 definitions inconsistently defines the relevant population. Consequently, we divided the neonatal ARDS patients who met the Montreux definition into two subgroups: those outside the perinatal period (> 7 d after birth), and the other within the perinatal period (≤ 7 d of birth).

When patients in the Montreux extra perinatal group were compared with those in the PALICC-2 group, no significant differences were observed in terms of morbidity in NICU, mortality, complications, or therapies. According to the PALICC-2 definition, OI or OSI was used to diagnose and stratify neonatal ARDS among infants receiving IMV, with OI used preferentially when available. Conversely, either the P/F or S/F ratio was used to diagnose and stratify neonatal ARDS for infants receiving NIV or HFNC (9). However, according to the Montreux definition, only OI can be applied to diagnose neonatal ARDS and to classify its severity, regardless of whether invasive or non-invasive ventilators were used (10). The outcomes between the cases in the Montreux extra perinatal group and the PALICC-2 group in the present study were similar, indicating that it is convenient and correct to use OI to diagnose neonatal ARDS and grade its severity in patients receiving IMV or NIV. Thus, the Montreux definition can be applied to evaluate the prognosis of all infants with neonatal ARDS.

There are some aspects of the PALICC-2 definition which do not apply to neonates. First, some of the clinical instruments and examination techniques used in the PALICC-2 definition are not commonly used in the NICU (i.e., cuffed endotracheal tubes, measured respiratory dead space, static compliance). Second, the S/F ratio and OSI in the PALICC-2 definition are not suitable for assessments of oxygenation in neonates due to wide variations in fetal hemoglobin concentrations that occur in neonates, frequent transfusion therapy, and uncertainty regarding the target SpO_2_ (25–27). In addition, the Montreux definition of neonatal ARDS involves diffuse and bilateral inflammation in the lungs, whereas the PALICC-2 definition advocates removal of the requirement of bilateral pulmonary infiltrates. To date, it remains controversial whether the definition of neonatal ARDS must include bilateral inflammation in the lungs. While the inclusion of bilateral pulmonary infiltrates on imaging in the definition of neonatal ARDS can distinguish the unique pathophysiology of ARDS from diseases such as lobar pneumonia or bronchiolitis (28), a chest radiograph is not sufficiently sensitive to detect all lung inflammation (29, 30). Furthermore, high variability among investigators may decrease the sensitivity of bilateral pulmonary infiltrates assessments (31, 32). Finally, the present study demonstrates that the Montreux definition may exclude some patients with neonatal ARDS due to the requirement that diffuse and bilateral inflammation in the lungs be observed. Therefore, it remains unclear whether the presence of bilateral pulmonary infiltrates is a necessary criterion for neonatal ARDS, and this requires further study.

Whether perinatal neonates should be included in the definition of neonatal ARDS remains controversial. In the present study, infants in the Montreux extra perinatal group were compared with infants in the Montreux intra perinatal group. Basic characteristics and triggers between these two groups were inconsistent, and the outcomes were quite different as well. Therefore, it is necessary to evaluate whether the definition of neonatal ARDS should include neonates in the perinatal period. To date, many studies have reported that the pathobiology of acute lung injury caused by perinatal events such as MAS, sepsis, infectious pneumonia, pulmonary hemorrhage, or perinatal asphyxia is similar to the diffuse inflammatory and injury mechanisms that characterize adult and pediatric ARDS (33–37). These observations support the perspective that ARDS may exist during the perinatal period. However, lung injury caused by perinatal events exhibit unique characteristics, and these are potentially related to postnatal lung morphogenesis, immune development, persistent fetal circulation, and changes in perinatal pulmonary vascular resistance (38). Therefore, triggers, morbidity, and prognosis of neonatal ARDS within the perinatal period differ from those outside the perinatal period. Similarly, there are differences observed between pediatric and adult ARDS cases in regard to infectious triggers, incidence, mortality, and prognosis (39–41). These differences emphasize that while neonates within the perinatal period may represent a special population, they should not be excluded from the definition of ARDS. In addition, further studies are needed to investigate whether neonatal ARDS caused by perinatal factors should be assessed by biopsy to confirm whether they are similar to the lung injuries caused by pediatric ARDS.

To the best of our knowledge, the present study is the first to compare the Montreux and PALICC-2 definitions in regard to neonates. Our results demonstrate that an advantage of the Montreux definition is that OI can be used to uniformly diagnose neonatal ARDS and grade severity independent of ventilation patterns. In addition, the Montreux definition specifies that acquired perinatal severe lung injuries should be included in the definition of neonatal ARDS, thereby making the Montreux definition more appropriate for diagnosing neonatal ARDS. However, there are also several limitations associated with the comparison performed in the present study that should be noted. First, while the present research was designed as a single-center, retrospective study, future research should include multicenter studies with larger sample sizes to evaluate the performance of the Montreux and PALICC-2 definitions in diagnosing neonatal ARDS and determining the stratification of case severity. Second, use of OI depended on accurate assessments of mean airway pressure (MAP) levels. At present, there are a greater number of patients who are receiving NIV in the NICU. The best approach for calculating MAP for these patients is to close their mouth with gentle pressure on the jaw and apply an interface of appropriate size, which is not compulsive in a NICU. Consequently, it remains difficult to achieve an accurate MAP level for patients receiving NIV, which influences the objectivity of the OI examined in this retrospective study. Third, previous studies have shown a strong correlation between OI and OSI (42), yet OSI is rejected by the Montreux definition. This may represent a limitation of the Montreux definition. We plan to further explore whether OSI can diagnose and grade neonatal ARDS when partial pressure of oxygen and transcutaneous oxygen pressure are not available. Lastly, there may be high variability among interobservers in regard to interpretation of chest imaging scans. Several studies have shown that lung ultrasound provides better accuracy for diagnosis of neonatal ARDS (43, 44). Thus, use of both lung ultrasound and chest radiographs may improve the efficiency of diagnosing neonatal ARDS in the future.

## 5. Conclusions

In conclusion, for infants outside the perinatal period that were examined in the present study, no significant differences in morbidity, mortality, severity, complications, or therapies were observed between the Montreux and PALICC-2 definition groups. For the neonates experiencing ARDS in the perinatal period, while this population may have a different pattern in epidemiology, clinical course, and prognosis, they exhibited a similar pathobiology pattern of ARDS (i.e., inflammation and cellular damage) at any age. Consequently, patients who are excluded by the PALICC-2 definition, yet are included based on the Montreux definition, should possibly not be excluded from a diagnosis of neonatal ARDS. Another advantage of the Montreux definition is that OI could be used to uniformly diagnose neonatal ARDS and grade severity independent of ventilation patterns. This paper is a single-center retrospective cohort study. As a result, selection and observation bias are present. Therefore, we suggest that further multicenter prospective cohort studies should be conducted to validate the present results and confirm a standardized definition of diagnostic criteria for neonatal ARDS.

## Data Availability

The original contributions presented in the study are included in the article/Supplementary Material, further inquiries can be directed to the corresponding author.
